# CFTIlandslides, Italian database of historical earthquake-induced landslides

**DOI:** 10.1038/s41597-024-03692-4

**Published:** 2024-08-02

**Authors:** Caterina Zei, Gabriele Tarabusi, Cecilia Ciuccarelli, Pierfrancesco Burrato, Giulia Sgattoni, Rita Chiara Taccone, Dante Mariotti

**Affiliations:** 1 Istituto Nazionale di Geofisica e Vulcanologia, Rome, Italy; 2 Università degli Studi di Ferrara - Dipartimento di Fisica e Scienze della Terra, Ferrara, Italy

**Keywords:** Natural hazards, Geology, Seismology

## Abstract

Knowing the location, the extent and the characteristics of any earthquake-induced environmental phenomenon is becoming an increasingly pressing need for civil protection agencies and local administrations. In particular, earthquake-triggered landslides are known for being among the most important sources of secondary hazard, as they may cause significant losses and may delay rescue operations across large areas. The combination of the relatively frequent seismic release with a very high landslide susceptibility makes the Italian territory especially prone to the occurrence of earthquake-induced landslides. The CFTIlandslides dataset features over 1,000 landslides triggered by historical Italian earthquakes (up to 1997). The landslides effects are subdivided into classes based on location accuracy and type of movement. Knowing the distribution of the past earthquake-induced landslides provides the input information for assessing the related hazard. This dataset is addressed to a large audience of potential users, including researchers and scholars, administrators and technicians belonging to local institutions, and civil protection authorities.

## Background & Summary

Among all European countries, Italy is by far the most widely affected by landslides. This is due to its physiography, featuring a great deal of high-relief landscape, and to the generally poor mechanical properties of the rocks exposed in the mountain chains. The official Italian landslide repository, *Inventario dei Fenomeni Franosi in Italia* (IFFI: https://www.progettoiffi.isprambiente.it/), lists all the known landslides that have occurred on the national territory according to standardized and shared methods. As of today, IFFI maps active landslides over an area of 7.9% of the national territory; therefore, over 5.5 million people live in areas of high landslide hazard^1^. But Italy, along with other southern European nations, also faces a high seismic hazard^2^. Both phenomena share a common origin in the complex geodynamic context of the central Mediterranean region, located along the boundary of the slowly converging Eurasian and Nubian tectonic plates. Over time, this activity has created geological and geomorphological predisposing conditions that make Italy prone to the occurrence of landslides at various scales. The earthquake ground shaking acts as a fundamental trigger for landsliding^3^; earthquake-induced landslides comprise a significant secondary co-seismic hazard^4^ and may subsequently cause additional cascading adverse phenomena^5^.

The database termed Catalogue of Strong Italian Earthquakes (hereinafter CFTI database) was compiled at the national scale and supplies a great deal of information and elaborations for all listed events^6^, including their effects on the social, built, and natural environments^7^; it holds a central role in assessing individual earthquake-induced landslide hazards, as its latest version (CFTI5Med)^8^ provides evidence of 527 effects of landslides that are known to have been induced by strong historical earthquakes. These *historical earthquake-induced landslides* (hereinafter HEILs) are the object of our investigation. HEILs are a subset of all landslides, but are generally not identified as earthquake-induced in official national landslide inventories such as IFFI.

In the framework of a collaboration between the CFTI Working Group and the CNR-IRPI, about 20 years ago, a first attempt was made to combine the available historical information on landslides caused by earthquakes with standard geomorphological techniques, including the interpretation of aerial photographs and field surveys, to better define the location, type, and distribution of HEILs^9^.

**Fig. 1:**
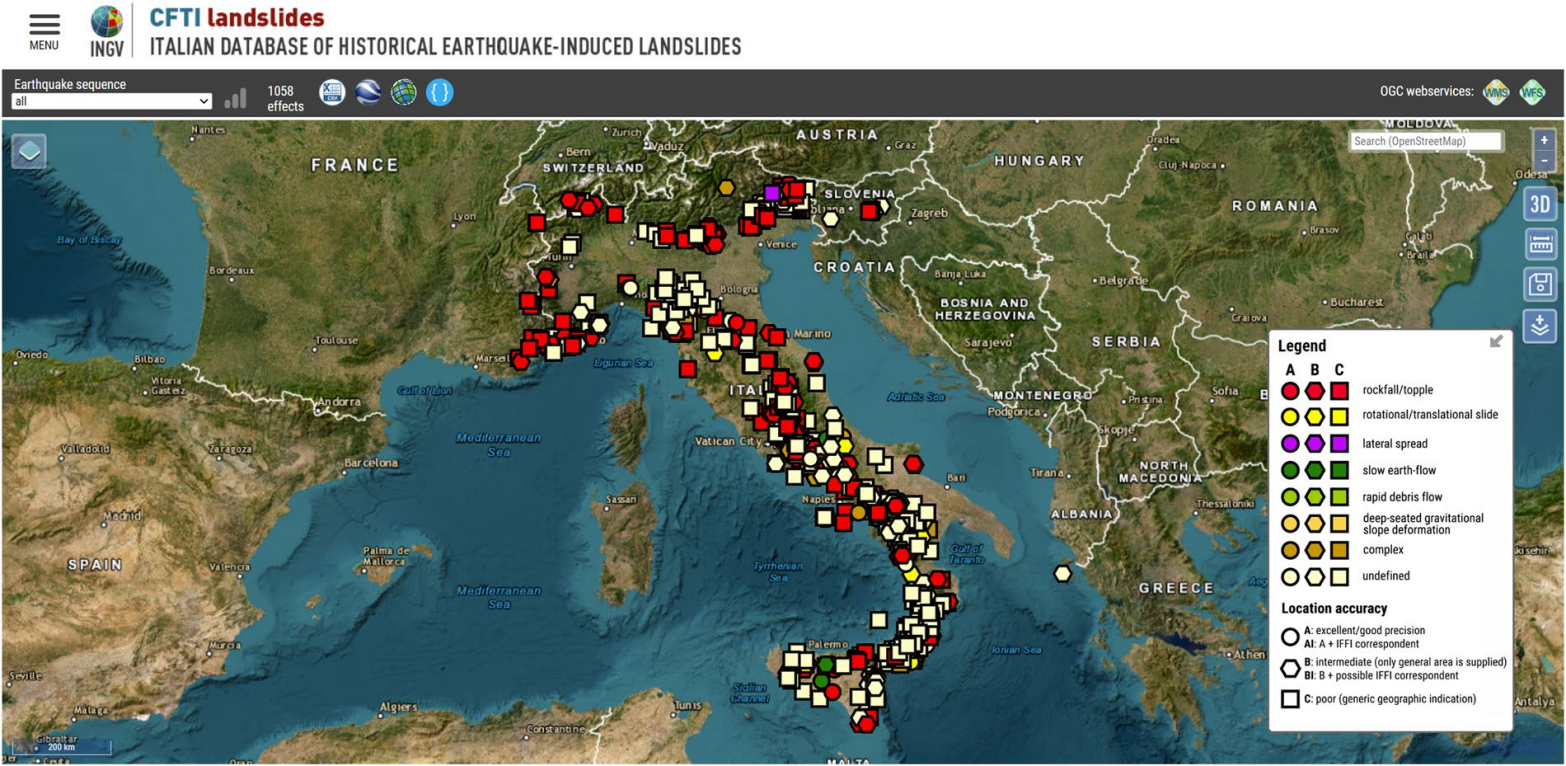
CFTIlandslides webGIS interface^10^.

In this work we reviewed and integrated the information already included in the CFTI database^6^ through the identification of new landslide effects, based on a review of literature and of historical sources; we then compiled a new dataset named CFTIlandslides, or the *Italian database of historical earthquake-induced landslides* (Fig. 1)^10^. We specifically focused on the analysis of newly found historical sources, or on the reappraisal of sources already known to the CFTI database^6^, of recent scientific articles and of technical reports. We also carried out a comparison with other digital archives such as the CEDIT^11^ (10.4408/IJEGE.2012-02.O-05) and the EEE^12^ (http://eeecatalogue.isprambiente.it/) catalogues. Our goal was to improve the location of each individual landslide effect and the definition of the nature of the causative landslide, whenever the descriptions of the historical sources allowed it. To this end we compared different types of datasets in a GIS environment, including aerial photographs, geomorphological maps and instability maps. Where possible, we linked the HEILs with individual landslides listed in the IFFI database.

We first included in our dataset a set of approximately 900 HEILs collected within the project “Multi-scale, integrated approach for the definition of earthquake-induced landslide hazard in Italy”, funded by the Italian Ministry for the Environment and completed in 2022. The goal of this project was to develop a multidisciplinary approach for assessing the earthquake-induced landslide hazard at national, regional, and local scales, and to integrate existing datasets with the results from previous projects and research activities. The main target of the investigations was the central Apennines region.

Over the past two years the activity continued by reviewing all earthquakes listed in the CFTI5Med for which landslide effects were reported, investigating them at different levels of detail (see Table 1).

**Table 1:** Ranking of landslide-triggering earthquake sequences based on the level of depth of data analysis.

Level of detail	Description	Number of earthquake sequences	Number of HEILs
High	All available and newly acquired historical sources were analyzed; the descriptions of the effects were improved or corrected.	30	443
Medium	There was a partial review of the available historical sources.	2	154
Low	Data were taken without any review of the descriptions supplied by CFTI5Med.	108	461

**Fig. 2:**
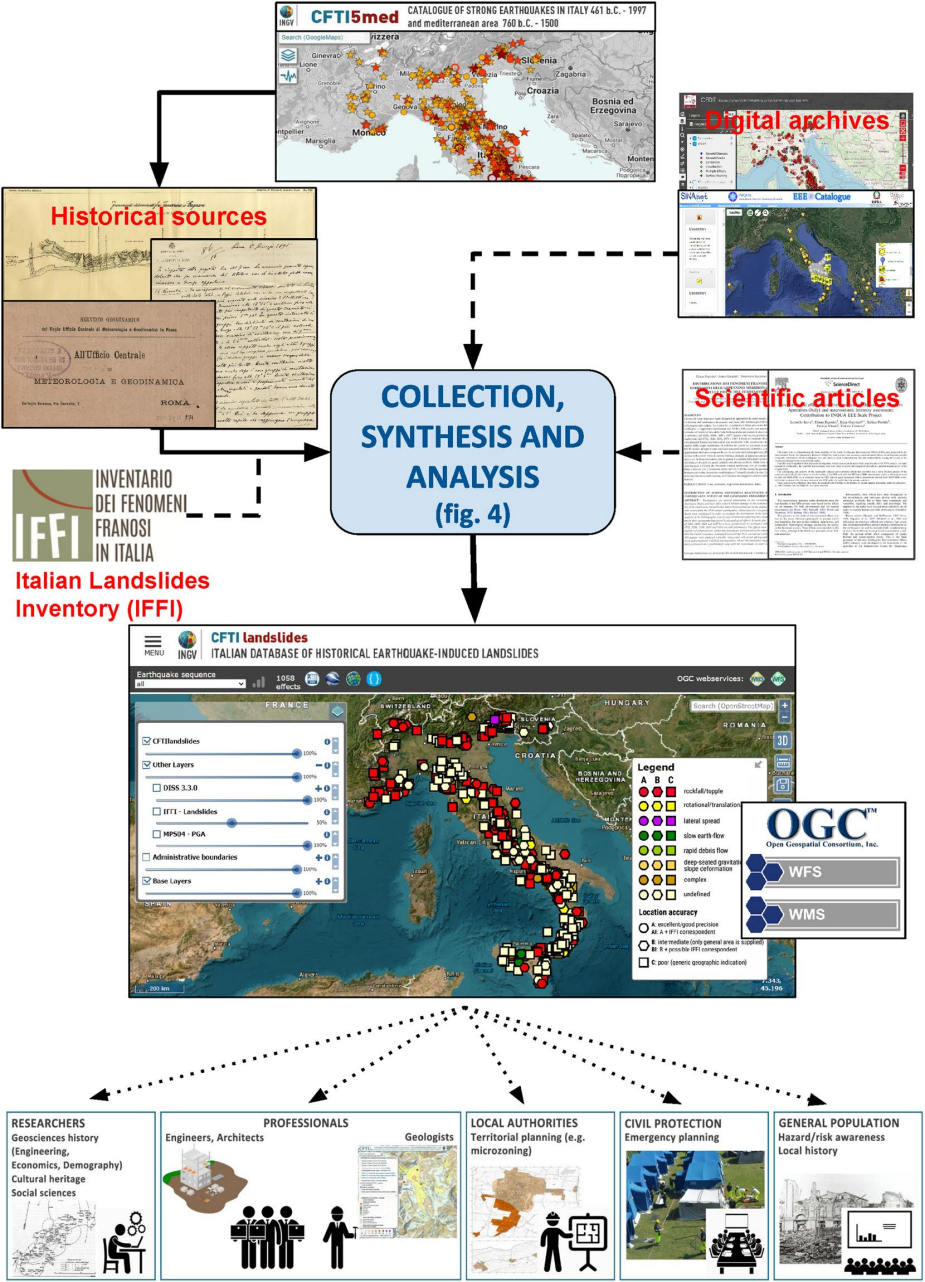
Workflow for the construction of the CFTIlandslides dataset^10^, starting from CFTI5Med and ending with the potential users and stakeholders.

The final result, forming the core of the first release of CFTIlandslides^10^, is a dataset of 1,058 landslide effects, each one linked to a specific earthquake listed by CFTI5Med; this new dataset is maintained by Istituto Nazionale di Geofisica e Vulcanologia (INGV) and is publicly accessible online at https://cfti.ingv.it/landslides (Fig. 1). It is addressed to a large audience of potential users and stakeholders, including researchers and scholars, administrators and technicians of local institutions, and civil protection authorities (Fig. 2).

Based on a review of literature and of historical sources, it is important to underline that there is no similar dataset of landslide effects on a global, regional, or national scale. The Comprehensive Global Database of Earthquake-Induced Landslide Events and Their Impacts^13^ (ver. 2.0, February 2022, 10.5066/P9RG3MBE), containing literature-documented earthquake-induced landslide events for the 249-year period from 1772 through August 2021, it is not a dataset of effects, but rather a collection of earthquakes that have caused landslides and the literature that describes them.

## Methods

**Fig. 3:**
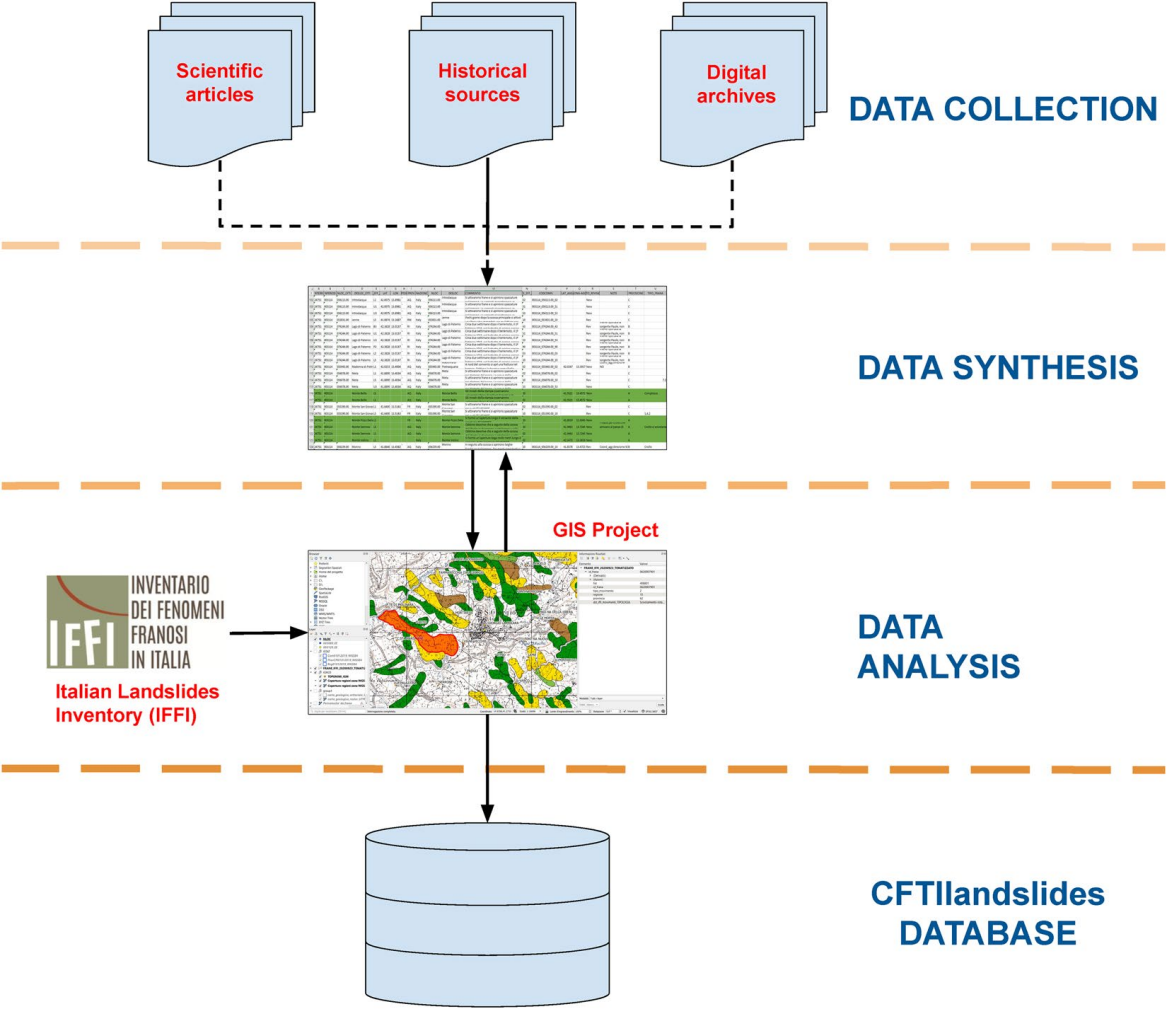
Workflow of our Collection, Synthesis and Analysis process (see Fig. 2).

The information contained in the CFTIlandslides^10^ dataset is based on a review of earthquake-induced effects on the environment originally supplied by the CFTI5Med earthquake catalogue (https://storing.ingv.it/cfti/cfti5/)^6,8^, improved and expanded using different sources of information (Fig. 3).

Since CFTIlandslides^10^ was conceived as a continuously updated repository, the earthquake sequences linked to the HEILs, were ranked based on the level of existing knowledge and depth of the analysis (Table 1). In any case, the one described here is the first version published and registered in INGV Data Registry. Future versions will have their own different DOI.

### Data collection and synthesis

The available historical information on earthquake-induced landslides is vast and documented by different hardcopy and digital files. Some of them consist of texts and iconography; in some instances, the effects were described by naturalists and geologists, while in other cases they appear in the news reported by ordinary witnesses or by newspapers. Due to the diverse and complex nature of the main information body, interpreting and summarising the different data into an organized table was a crucial part of the work.

To reach this goal, the revision proceeded through three subsequent steps:

1. *Review of historical sources*, newly found or already archived in the CFTI database^6^.
  We analysed various types of documents, including texts, letters, reports, newspapers, images, photographs, and maps (Fig. 4). Over 800 documents obtained from public, private and online libraries and archives were reviewed. Of these, more than half contained useful information. A meticulous analysis of the sources allowed us to collect new landslide evidence and more detailed descriptions of landslide effects already included in the CFTI database^6^.

**Fig. 4:**
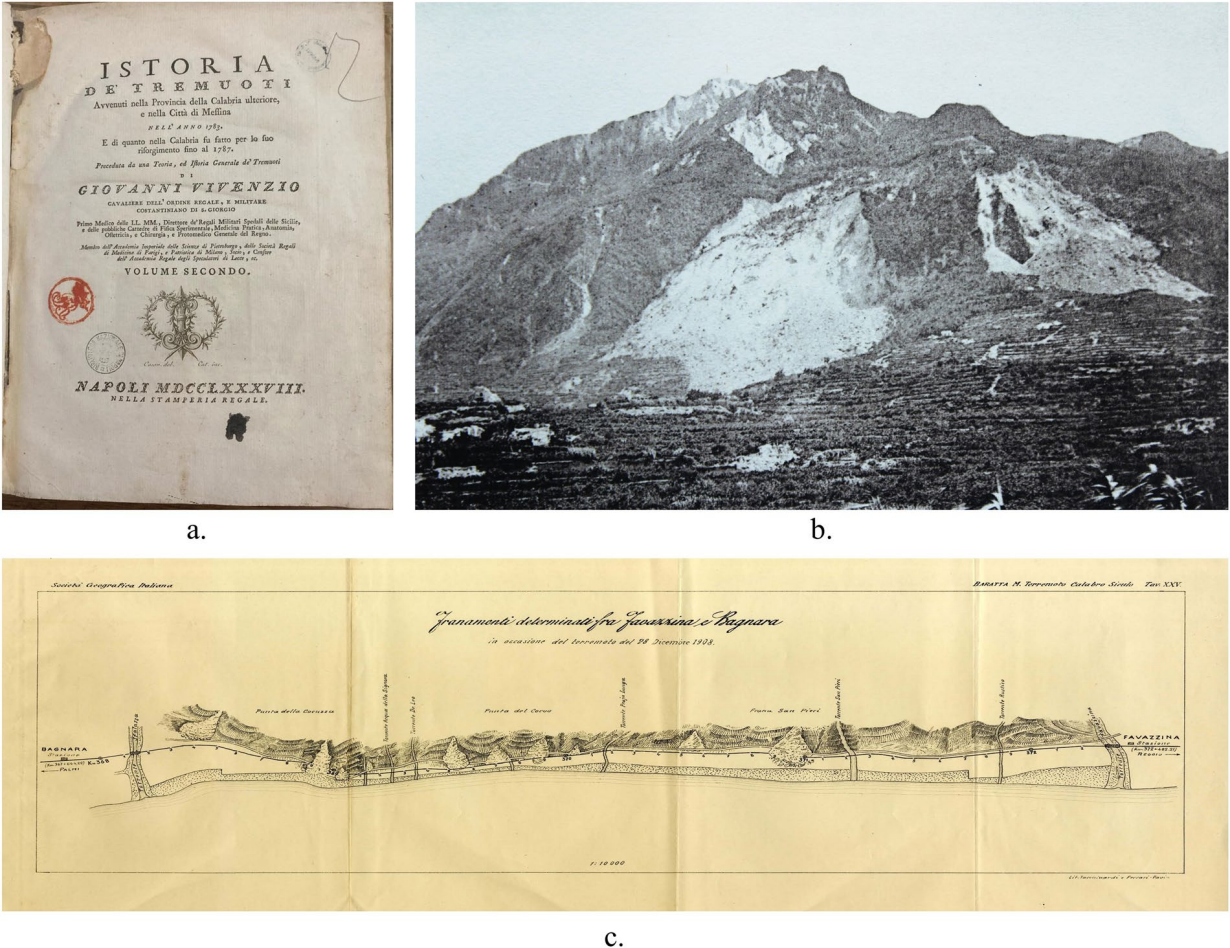
Examples of historical sources. (**a**) Original historical report on the 1783 Calabria earthquakes^16^; (**b**) Photograph of the Monte Epomeo landslide following the 1883 Isola d’Ischia earthquake (*Johnston-Lavis UCL Geology Collection*)^17^; (**c**) Map of landslides that occurred between Favazzina and Bagnara following the 1908 Messina Straits earthquake^18^.
2. *Analysis of recent scientific articles and technical reports*.
  We collected and examined the latest scientific literature describing earthquake-induced effects in the Italian territory, in search of new evidence arising from historical sources unknown to the CFTI database^6^. We gathered 569 bibliographical references, including historical sources, scientific articles and technical reports, all of which contained information about earthquake-induced landslides; they are listed in the bibliography of CFTIlandslides^10^. All these references are stored in the CFTI database^6^ with a unique identification code; 436 of them are publicly available in PDF format and can be downloaded through the CFTIlandslides website^10^.
3. *Comparison with other digital archives*.

We searched the CEDIT^11^ (10.4408/IJEGE.2012-02.O-05) and the EEE^12^ (http://eeecatalogue.isprambiente.it/) catalogues for any description of HEILs not already included in our dataset. For each retrieved effect we verified the original reference, the information available and the description.

After completing these three steps, we combined and summarised the data collected from different sources into a single text file. The information was archived based on the landslide place name. Under each location we reported the identification code of each historical source and the associated original HEIL descriptive text. This step allowed us to represent the entire dataset in a table format, containing all relevant information and the preliminary geographic location of each HEIL (see Data Record section).

### Data analysis

The geographic information supplied by the historical sources allowed us to locate the effects of each individual landslide, at least preliminarily, and display it in a GIS environment; especially detailed and exhaustive descriptions often allowed to improve this location substantially. Each landslide effect was assigned its best possible location using satellite images, historical and topographic maps, and toponymy maps.

**Fig. 5:**
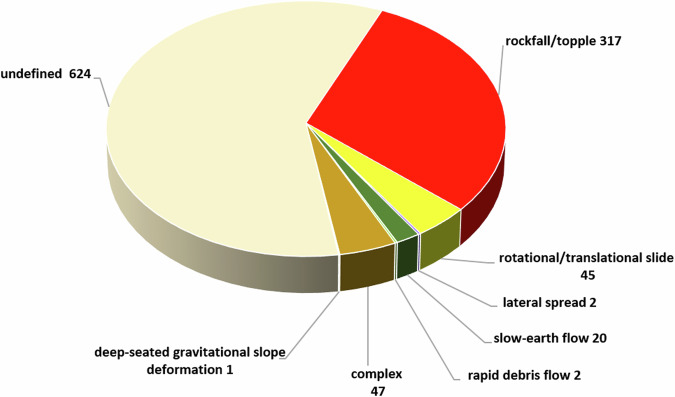
Distribution of earthquake-induced landslides by type of movement.

When the texts of the historical sources allowed us to characterise the type of movement of a landslide, we followed the classification adopted by the IFFI database (Fig. 5).

**Fig. 6:**
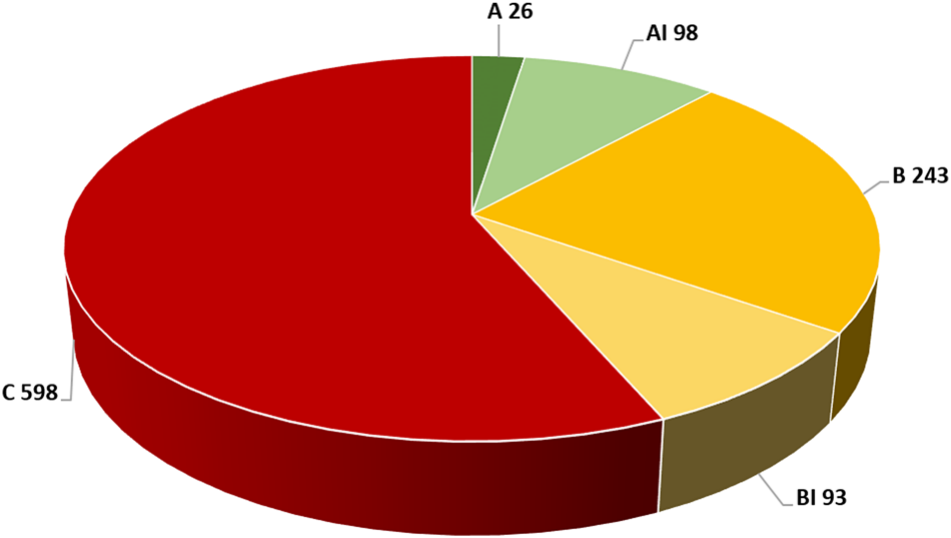
Distribution of earthquake-induced landslides by location accuracy (see text for the definition of each accuracy class).

Following the location analysis, the landslides effects were subdivided into three classes according to location accuracy (Fig. 6):

- **Class A**: the effect is well located geographically through an unambiguous toponym, a detailed indication, or a name. Examples of description are “*…on the left corner of Casa Baroncioni…*” or “*…at km 56 of the provincial road…*”;
- **Class B**: the effect falls in an identified broad area, but no further specification is available. Examples of such descriptions are “*…on the ground near the northwestern part of the village…*” or “*…along the railway between Scilla and Favazzina…*”;
- **Class C**: the effect is associated with a locality of the CFTI database^6^, but no specific geographic indication is available.

The assignment to one of the three classes is based on the quality of the historical information and on the possibility of identifying the site of the effect on modern maps; it is not a metric value.

Moreover, the location of each class A and B landslide effect was compared with the location of all individual landslides included in the Italian Landslide Inventory (IFFI database)^14^: when a satisfactory geographical correspondence with an IFFI landslide was found, the two items were paired.

For this specific case we created two additional classes:

- Class AI: if a Class A record is associated with one of the landslides included in IFFI;
- Class BI: if a Class B record is associated with one of the landslides included in IFFI.

The type of landslide movement described in the historical sources is almost always consistent with the landslide properties supplied by IFFI, except for two recurring cases:

- lack of information from the historical sources: in this case, we adopted the landslide typology of the associated IFFI record;
- following the corresponding IFFI record, we reported as complex some landslides described as rockfalls by historical sources.

The table-format dataset and the GIS project were conceived to collect the information and consult HEILs individually and on a map. These two products were mutually connected to improve data quality. When a landslide toponym mentioned by the historical sources is found on the GIS project map, the associated coordinates are reported on the table-format dataset. Otherwise, if the toponym of the historical landslide has changed and contemporary maps report a new place name, the table-format dataset is updated accordingly.

### CFTIlandslides dataset

The dataset of the CFTIlandslides^10^ is published with a Creative Commons Attribution 4.0 International license (CC BY 4.0). Currently it includes 1,058 landslides that can be consulted and downloaded from a dedicated INGV website (10.13127/cfti/landslides), also accessible from the recently renewed CFTI portal by Istituto Nazionale di Geofisica e Vulcanologia (https://cfti.ingv.it)^15^. The metadata are also archived in the INGV Data Registry, publicly accessible through the Open Data Portal of Istituto Nazionale di Geofisica e Vulcanologia (https://data.ingv.it/dataset/964).

**Fig. 7:**
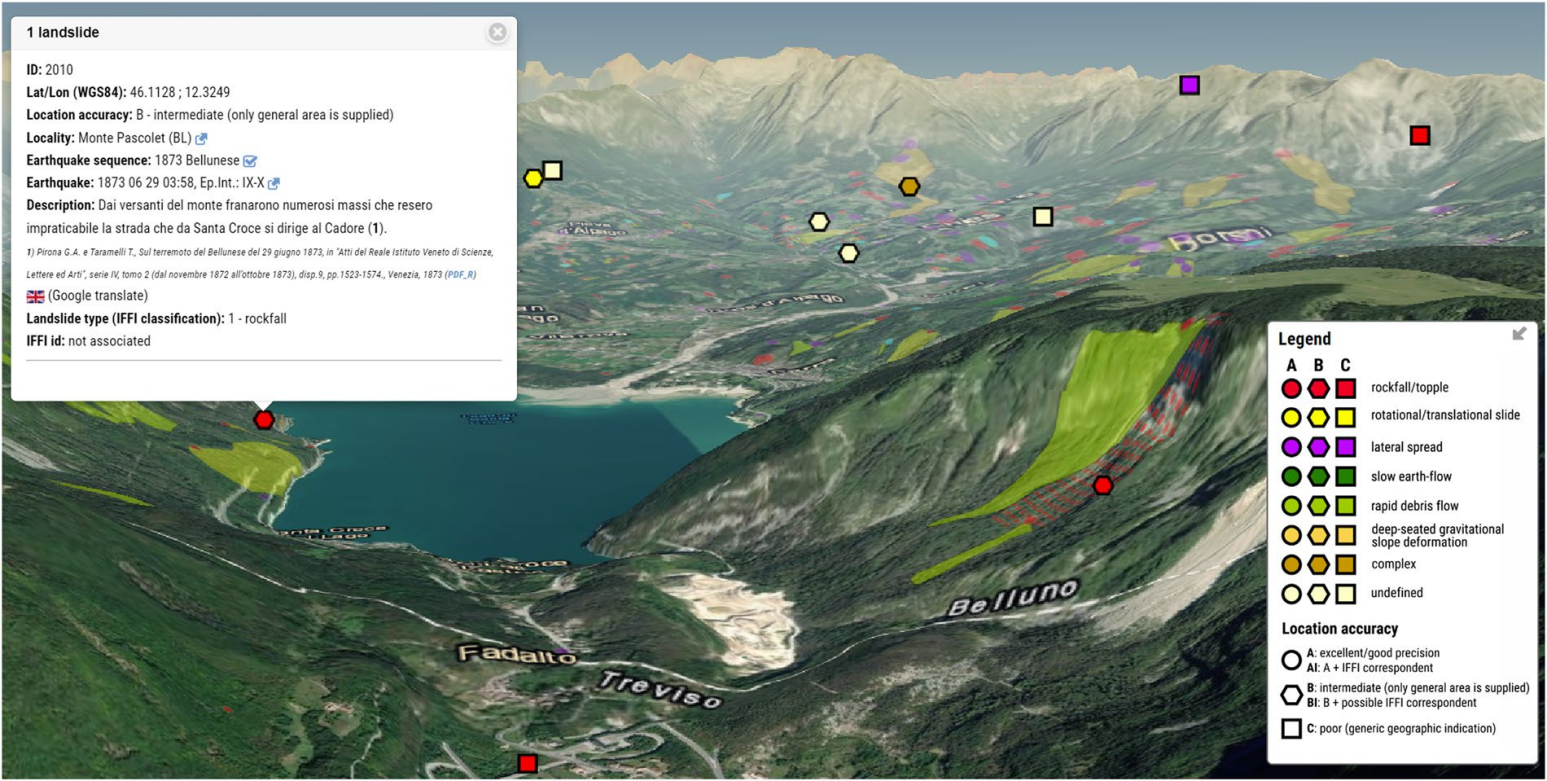
The CFTIlandslide WebGIS application allows data consultation also on a 3D terrain map. Here the effects of earthquake-induced landslides are shown over an orthophoto map along with the relief of the digital terrain model and compared with IFFI landslides data (via ISPRA WMS service). An example of the complete data sheet available for each landslide effect is shown at the top left.
- Open Geospatial Consortium (https://www.ogc.org/) web services (Table 2).

To make CFTIlandslides^10^ data publicly available through the website we developed:

- a custom WebGIS application (Figs. 1, 7);

**Table 2:** OGC Web Services available for the CFTIlandslides dataset.

Service type	Definition	Url
Web Feature Service (WFS)	Supports requests for geographical feature data (with vector geometry and attributes).	https://cfti.ingv.it/geoserver/CFTIlandslides/wfs?service=WFS&request=getCapabilities
Web Map Service (WMS)	Supports requests for map images (and other raster formats) generated from geographical data.	https://cfti.ingv.it/geoserver/CFTIlandslides/wms?service=WMS&request=getCapabilities

Through the OGC web services, data can also be downloaded as CSV (Comma-Separated Values), SHP (Esri shapefile), KML (Keyhole Markup Language), or GeoJson files.

## Data Records

The CFTILandslides^10^ dataset stores information on 1,058 HEILs that occurred in Italy from 117 B.C. to 1997, associated with 140 different earthquake sequences. It is published online and registered in the INGV Data Registry with a DOI (Digital Object Identifier) (10.13127/cfti/landslides) to provide a persistent identification. It consists of two main files that can be directly downloaded via the links in brackets:

- **Landslide_effects table**

It supplies all 1,058 known earthquake-induced landslide effects: it contains the following fields:

1. **Id_LS**: Identification code of the data record (primary key).
2. **Lat**: Latitude north in decimal degrees within the WGS_1984 geographic coordinate system, rounded to four decimal places.
3. **Lon**: Longitude east in decimal degrees within the WGS_1984 geographic coordinate system, rounded to four decimal places.
4. **Id_eq**: ID of an Individual Earthquake in the CFTI database^6^ (external key). [only if the association is made possible by the accuracy of the testimonies]
5. **Id_seq**: ID of an Individual Earthquake Sequence in the CFTI database (external key).
6. **Id_loc_CFTI**: ID of a locality of the CFTI database^6^ (external key).
  Locality: (from CFTI database^6^);
  Province: (from CFTI database^6^);
  Nation: (from CFTI database^6^)
7. **Location_accuracy**: Class of location accuracy (A, B, or C).
8. **Landslide_Type**: Landslide type (IFFI classification).
9. **Id_IFFI**: IFFI identification code (external key). [only if the association is made possible by the data analysis]
10. **Description_ITA**: Description of landslide effects (original, in Italian).
11. **Description_ENG_google**: Description of landslide effects (translation in English, based on Google Translate).

- **EqSeq_Catalogue Table**

It supplies the list of the earthquake sequences for which landslide effects are known and consisting of the following fields:

1. **Id_seq**: ID of an Individual Earthquake Sequence in the CFTI database^6^ (primary key).
2. **Year**: Year when the earthquake sequence took place.
3. **Area**: Area of the main effects of the earthquake.
4. **LR_ls**: Level of review of the landslide effects of the Earthquake Sequence (low, medium, high); see Table 1.

The connection between the two tables is based on the Earthquake Sequence IDs (Id_seq).

For both tables, external keys allow SQL joins with the CFTI database^6^ to retrieve data about individual earthquakes and localities.

External keys also allow linking directly from the CFTIlandlides website (10.13127/cfti/landslides) to the individual earthquake page of CFTI5med^8^ and the individual landslide page of the IFFI database.

## Technical Validation

The CFTILandslides^10^ dataset contains data on landslides induced by historical earthquakes. It has already been highlighted^6^ that due to their “randomly regular” occurrence pattern, historical earthquakes do not allow for formal data validation through any experiment.

At any rate, the reliability and technical quality of the CFTILandslides^10^ dataset are ensured by its characteristics of data homogeneity, transparency, and accuracy of classification.

The homogeneity of the data is guaranteed:

- by the information being exclusively based on primary historical sources, i.e. texts that provide direct, coeval, or first-hand evidence about each landslide. These sources are critically analysed, compared, and interpreted with the techniques of the historical method. Data from other datasets (CEDIT^11^ and EEE^12^ catalogues) and literature are also acquired, but only if the primary historical sources that mention them can be retrieved;
- by the rigorous interpretative criteria, which do not involve assumptions or hypotheses regarding the communicative intentions of the sources used. A record is created only if the primary sources explicitly refer to ground movement (even with a single word), taking into account the linguistic peculiarities of different periods and places;
- by the rules established for formulating the data synthesis: they require specifying the location, the precise phenomenology of the event and its dating, or explicitly indicating if such information is missing in the sources.

In addition to guaranteeing data homogeneity, these criteria allow for the control and avoidance of possible duplications and overinterpretations of the records.

Regarding the transparency of CFTIlandslides^10^ data, it should be pointed out that this dataset provides access to the basic data (the primary sources), thus enabling users to critically re-evaluate the data critically; either directly, through links to the text of historical sources, or indirectly, through their references. Data processed with these criteria of homogeneity and transparency possess a high level of accuracy and reliability.

Furthermore, the classification of location reliability allows for handling the data accuracy statistically.

The association of HEILs with those reported in the IFFI database, which is based on international standards of classification and nomenclature and on a comprehensive record, allows for useful verification opportunities and makes it possible to add data to a specific earthquake-induced landslide.

## Usage Notes

The custom code used to develop the CFTIlandslides^10^ web interface is entirely open and based on HTML language. As such, it can be reutilised by whoever may be interested in replicating our experience elsewhere.

The server-side procedures were developed in PHP open-source language. The client-side procedures were developed in JavaScript language, using the open-source library OpenLayers (https://openlayers.org/) and its extensions “OL-ext” (https://viglino.github.io/ol-ext) to provide a reliable and fast geographic interface, and “OL-Cesium” (https://openlayers.org/ol-cesium/) for 3D map visualization.

## Data Availability

No custom code has been used to curate the dataset.
